# Impact of irradiance and inorganic carbon availability on heterologous sucrose production in *Synechococcus elongatus* PCC 7942

**DOI:** 10.3389/fpls.2024.1378573

**Published:** 2024-04-08

**Authors:** Lisa Yun, Robert Zegarac, Daniel C. Ducat

**Affiliations:** ^1^ Department of Biochemistry and Molecular Biology, Michigan State University, East Lansing, MI, United States; ^2^ Department of Energy-Michigan State University Plant Research Laboratories, Michigan State University, East Lansing, MI, United States

**Keywords:** cyanobacteria, sucrose, feedstocks, bioproduction, high cell density, cultivation

## Abstract

Cyanobacteria have been proposed as a potential alternative carbohydrate feedstock and multiple species have been successfully engineered to secrete fermentable sugars. To date, the most productive cyanobacterial strains are those designed to secrete sucrose, yet there exist considerable differences in reported productivities across different model species and laboratories. In this study, we investigate how cultivation conditions (specifically, irradiance, CO_2_, and cultivator type) affect the productivity of sucrose-secreting *Synechococcus elongatus* PCC 7942. We find that *S. elongatus* produces the highest sucrose yield in irradiances far greater than what is often experimentally utilized, and that high light intensities are tolerated by *S. elongatus*, especially under higher density cultivation where turbidity may attenuate the effective light experienced in the culture. By increasing light and inorganic carbon availability, *S. elongatus cscB*/*sps* produced a total of 3.8 g L^-1^ of sucrose and the highest productivity within that period being 47.8 mg L^-1^ h^-1^. This study provides quantitative description of the impact of culture conditions on cyanobacteria-derived sucrose that may assist to standardize cross-laboratory comparisons and demonstrates a significant capacity to improve productivity via optimizing cultivation conditions.

## 1 Introduction

There is a need for sustainable carbohydrate feedstocks as we increasingly look to biotechnology and green chemistry approaches for energy and other commodity chemicals. Sucrose is one significant feedstock for bio-ethanol (Balat and Balat, 2009) and is a promising building block for many value-added chemicals (Peters et al., 2010). However, sucrose is typically produced by plant crop species (*e.g.*, sugar beet and sugar cane) (Peters et al., 2010), giving rise to ethical concerns about diverting arable land and potable water from food production to fuel and chemicals (Graham-Rowe, 2011; Lang et al., 2017). Cellulosic biomass is another promising alternative source of fermentable carbohydrates that is under active study, yet the high costs of converting lignocellulosic materials into simple carbohydrates suitable for fermentation is currently prohibitive (Graham-Rowe, 2011; Ziolkowska, 2014). For these reasons, an alternative source for sucrose that circumvents the controversies and complexities of plants is desirable.

Cyanobacteria are a diverse phylum of photosynthetic prokaryotes that are under consideration as an alternative carbohydrate feedstock species due to their ability to hyperaccumulate soluble sugars under certain growth conditions (Hays and Ducat, 2015). Moreover, cyanobacteria are less likely to compete for land and water resources with food crops, are faster growing, relatively easier to genetically engineer, and have a higher photosynthetic efficiency than land plant sources of carbohydrate feedstocks (Parmar et al., 2011; Satta et al., 2023). One promising approach for cyanobacterial sucrose production involves the heterologous expression of sucrose permease (*cscB*), which was originally reported to allow up to ~80% of fixed carbon to be exported in the form of sucrose when expressed in the model cyanobacterium, *Synechococcus elongatus* PCC 7942 (Ducat et al., 2012). The high productivity of such strains is in part due to an emergent property whereby photosynthetic activity is increased: exporting sucrose increases the quantum efficiency of photosystem II, rate of photosystem II oxygen evolution, and carbon fixation rate (Ducat et al., 2012; Abramson et al., 2016; Santos-Merino et al., 2021, 2023; Wang et al., 2023). Additional genetic modifications have been employed to increase sucrose export by increasing carbon flux to the sucrose synthesis pathway and/or decreasing flux to potential competing pathways, with mixed success (Santos-Merino et al., 2023). For example, multiple groups have shown that sucrose productivity can be enhanced by artificially over-expressing sucrose phosphate synthase (*sps*), an enzyme involved in a rate-limiting step of sucrose synthesis, in the background of sucrose exporting lines (Ducat et al., 2012; Duan et al., 2016; Hays et al., 2017; Qiao et al., 2018).

Similar strategies to enable sucrose export have been used across a variety of cyanobacterial species; *cscB* has been expressed in *S. elongatus* UTEX 2973 (Lin et al., 2020; Zhang et al., 2020), *Synechococcus* sp. PCC 7002 (Xu et al., 2013; Han et al., 2023), and *Synechocystis* sp. PCC 6803 (Du et al., 2013; Thiel et al., 2019). Different cyanobacterial species exhibit varying rates of sucrose productivity (Santos-Merino et al., 2023), though it is often uncertain if inter-species differences are due to genetics, distinct conditions utilized for laboratory growth, metabolic requirements, tolerances for environmental stresses, or inherent photosynthetic capacities (Billis et al., 2014; Ungerer et al., 2018b; Cassier-Chauvat et al., 2021; Adomako et al., 2022). For instance, *S. elongatus* PCC 7942 and *S. elongatus* UTEX 2973 share 99.9% genome similarity, yet the latter has significantly faster growth rates, higher light and temperature tolerances, and has been reported to have a significantly higher sucrose productivity (Yu et al., 2015; Ungerer et al., 2018b; Lin et al., 2020; Adomako et al., 2022). A handful of single nucleotide polymorphisms appear to significantly contribute to enhanced tolerance of light and heat in *S. elongatus* UTEX 2973 (Ungerer et al., 2018b), and these strains also exhibit substantially faster growth rates (2.5 hour doubling time compared to 7-8 hours). Therefore, it is possible that the increased sucrose productivity of engineered *S. elongatus* UTEX 2973 strains may be due to an inherently improved photosynthetic capacity. Alternatively, other factors like higher routine temperature, light growth conditions or altered partitioning of fixed carbon may contribute to the productivity of *S. elongatus* UTEX 2973 (Mueller et al., 2017; Hendry et al., 2019).

In this study, we set out to systematically assess how different laboratory equipment and growth conditions impact the efficacy of sucrose production, with a particular focus on light and inorganic carbon availability as two critical factors that may impact sucrose secretion rates, and that could contribute (in part or in full) to the differences in sucrose productivity observed across different laboratories and cyanobacterial species. The interest in exploring cyanobacteria as an alternative carbohydrate feedstock has led to many recent reports documenting sucrose production, yet, as we recently reviewed (Santos-Merino et al., 2023), there can be considerable variation in productivity between different publications, even when the species and genetic modifications are identical. We explored a variety of commercial and custom-built cyanobacterial growth chambers, and report cultivation conditions and bioproduction values in standardized units to facilitate cross-comparisons between laboratories. We find a direct relationship between irradiance used for cyanobacterial cultivation and sucrose productivity, especially when inorganic carbon availability was not limiting. Our results may be valuable for the broader community of scientists working with different strains of cyanobacteria engineered as potential alternative carbohydrate feedstocks, and especially point to limitations imposed on productivity caused by inefficient CO_2_ delivery methods. Additionally, our results have implications for the degree of variation in sucrose productivity of related cyanobacterial species, which may be significantly influenced by cultivation conditions rather than solely due to inherent metabolic and physiological limitations of slower-growing species.

## 2 Materials and methods

### 2.1 Strains and cultivation conditions

The base strain of cyanobacteria we utilize for our sucrose measurements is constructed as reported in Abramson et al., 2016. Briefly, this strain contains a copy of sucrose permease (*cscB*) under an IPTG-inducible Ptrc promoter installed into Neutral Site 3. An additional copy of sucrose phosphate synthase (*sps*) cloned from *Synechocystis* sp. PCC 6803 is also expressed under Ptrc and is integrated into the genome at Neutral Site 2. Inducible *sps* allows us to bypass the requirement for external salt for sucrose production that is in many sucrose-secreting strains. For results from “wild-type” (WT) strains, we use the ATCC strain of *S. elongatus* PCC 7942 (ATCC #33912).

*S. elongatus* PCC 7942 strains were routinely grown in BG-11 media (C3061, Sigma-Aldrich, USA) supplemented with 1 g L^-1^ HEPES and set to pH 8.3 with NaOH to maintain or expand cultures. Cultures were incubated in a Multitron Infors HT incubator at 32°C with 2% CO_2_, 150 rpm shaking, and ~150 μmol photons^-2^ s^-1^ of continuous light from Sylvania 15 W Gro-Lux fluorescent bulbs. To standardize the growth history of strains between different experiments, all cultures were back-diluted 1:10 into BG-11 for three days prior to initiating any of the reported experiments. When needed, 1 mM IPTG was added to induce the expression of *cscB* and *sps* genes at time = 0 h. Chloramphenicol (Cm; 25 μg mL^-1^) and kanamycin (Kn; 50 μg mL^-1^) were used to maintain *cscB* and *sps*, respectively. Antibiotics were not added on the final day of back-dilution to minimize confounding effects.

### 2.2 Examining light tolerance and sucrose production in MC-1000 multi-cultivators

The MC-1000 multi-cultivator system (Photon System Instruments, Czech Republic) was used to examine the light tolerance of *S. elongatus* PCC 7942; the device emits cool white light at varying light intensities (150, 250, 500, 1000, 2000, or 2500 μmol photons m^-2^ s^-1^). MC-1000 cultures were kept at 32°C and provided with 3% CO_2_. Overnight cultures were back-diluted to an OD_750_ of ~0.3 into MC-1000 vessels with fresh BG-11 media supplemented with 10 mM HCO_3_^-^, and induced with 1 mM IPTG as needed for low-starting density experiments. Samples were taken at 0, 24, 48, and 72 h for OD_750_ and sucrose measurements.

For the experiments specifically evaluating high-light with high-starting density cultivation in the MC-1000 system, overnight cultures (prepared as outlined in section 2.1) were pelleted in a benchtop centrifuge. The supernatant was removed, and the cell pellet was resuspended with fresh BG-11 media supplemented with 10 mM HCO_3_^-^ to an OD_750_ of ~1.5 or 2.0 (provided 2000 and 2500 μmol photons m^-2^ s^-1^, respectively). An additional 10 mM HCO_3_^-^ was added at 24 h for vessels that were provided bicarbonate daily. Samples were taken at 0, 24, and 48 h for OD_750_ measurements and sucrose quantification.

### 2.3 Sucrose production in high-density multi-cultivators

The High-Density Cultivation (HDC) 6.10B starter kit (CellDEG GmbH, Germany) was used for high-CO_2_ and high-density cultivation. The kit consists of a bottom base with a buffer reservoir and 10 mL cultivators with a gas-permeable bottom membrane. At the start of the experiments, the reservoir was filled with 200 mL of a 3 M KHCO_3_/3 M K_2_CO_3_ solution to provide CO_2_. Cultures prepared in BG-11 were centrifuged for 10 min at 3700 x*g* and 22°C before removing the supernatant and resuspending to an OD_750_ of ~0.3 with CD media. CD media is designed to sustain extended periods of high-density cultivation of photosynthetic microbes, and has higher concentrations of macronutrients (N, P, and K) and micronutrients (Mg, S, Ca, Na, Cl, Fe, Zn, and Mo) relative to BG-11. Due to its enrichment, CD media has a high osmolarity (~140 mOsm) that is approximately three times greater than of BG-11 (~40 mOsm); the exact composition is available on protocols.io (dx.doi.org/10.17504/protocols.io.2bxgapn). Relevant cultures were induced with 1 mM IPTG before transferring 12 mL to the HDC system cultivators. Moderate-light HDC experiments were incubated in a Multitron incubator (Infors HT, Switzerland) providing ~150 rpm shaking and ~250 μmol photons m^-2^ s^-1^ from Sylvania 15 W Gro-Lux fluorescent bulbs. High-light HDC experiments were incubated in a growth chamber (AL-36L4, Percival Scientific, USA) with either ~500 μmol photons m^-2^ s^-1^ for 0-120 h, or ~500 μmol photons m^-2^ s^-1^ for 0-48 h and ~1000 μmol photons m^-2^ s^-1^ for 48-120 h from a light array fabricated in-house consisting of four equidistant chip-on-board (COB) LED lights (BXRA-56C5300-H, Bridgelux, USA)(**Supplementary Figure S1**). Both incubators provided 2% ambient CO_2_, constant illumination, and ~150 rpm shaking. Samples were taken at 0, 24, 48, 72, 96, and 120 h to measure OD_750_ and to collect supernatant for sucrose quantification.

### 2.4 Measuring light penetration through culture depth

The following experimental configuration was used to measure the relationship between surface irradiance, light penetration, and culture turbidity: a glass graduated cylinder with a clear bottom was clamped onto a ring stand. A Submersible Spherical Micro Quantum Sensor (US-SQS/L; WALZ, Germany) connected to a LI-250A light meter (LI-COR, USA) was clamped above and in-center of the graduated cylinder. A single COB LED from the in-house lighting device was placed below and center of the graduated cylinder. With this setup, 5 cm of liquid (BG-11 or cell culture at the indicated OD_750_) fill the graduated cylinder, and the sensor measures the light that penetrates through the liquid when 0, 1, 2, or 3 cm away from the light interface of the culture (0 cm). A black, opaque sheet was wrapped around the graduated cylinder to cover all but the bottom 4 cm of the culture to block ambient light.

To set the desired irradiance (150, 500, 1000, 2000, 3000, or 4000 µmol photons m^-2^ s^-1^), the graduated cylinder was filled with 5 cm of BG-11, and the sensor was placed flush to the bottom of the glass to measure the incoming light at 0 cm. WT cultures were prepared as outlined in Section 2.1, pelleted, and supernatants removed. Pellets were resuspended with fresh BG-11 to create a concentrate that was diluted with BG-11 to 0.3, 1.0, 2.0, 5.0, and 10.0 OD_750_. Five mL of each culture filled the graduated cylinder and penetrating irradiances at 1, 2, and 3 cm were recorded at applicable light intensities. For this and setting the irradiance, the average function of the LI-250A was used in which the device averages the values recorded over a 15-second interval.

### 2.5 Sucrose quantification

Samples collected at the time points specified above were pelleted in a microcentrifuge; the supernatants were saved and stored at -20°C until the end of the experiment. Sucrose quantification was performed using the Sucrose/D-Glucose Assay Kit (K-SUCGL; Megazyme, USA).

### 2.6 Dry cell weight determination

To determine the dry cell weight of *S. elongatus* PCC 7942 strains, cultivation conditions were recreated as outlined in Section 2.2 for low-starting density experiments in the MC-1000, and Section 2.3 for moderate-light experiments in the HDC. Samples were collected at a variety of culture densities (OD_750_) in order to generate a broad distribution for standard curves correlating OD_750_ with dry cell biomass (**Supplementary Figure S2**). The OD_750_ of collected samples was determined with a visible spectrophotometer (Genesys 20; Thermo Fisher Scientific, USA), washed with Milli-Q water twice, and resuspended into the desired OD_750_ with Milli-Q water. Three to 20 mL of the prepared samples were pelleted in a Falcon tube and most of the supernatant removed; the remaining water was used to create a concentrated resuspension to transfer to a pre-weighed 3 mL glass test tube. Milli-Q water was used as needed to rinse the Falcon tube and added to the glass test tube. Samples were dried in a 90°C oven for 24 h, or until the mass remained stable. The mass of the empty glass test tube was subtracted from the final weight, and the resulting value was divided by the volume used to determine the dry cell weight (g L^-1^) for corresponding OD_750_. Python was used to create a linear regression model and derive the function parameters, which was then used to convert OD_750_ into dry cell weight.

## 3 Results

### 3.1 Assessing high-light tolerance in *S. elongatus*

We first selected a strain of *S. elongatus* PCC 7942 that has been engineered for sucrose secretion in a salt-independent manner. Briefly, hyperaccumulation of cytosolic sucrose is a common strategy for osmotic stress protection in many freshwater cyanobacterial species (Klähn and Hagemann, 2011), where this compatible solute can act as a counter osmoticum while also conferring other cellular protective effects. A rate-limiting step of sucrose biosynthesis occurs at the enzyme sucrose phosphate synthase (SPS), that catalyzes the condensation of NDP-glucose with fructose-6-phosphate to form sucrose-6-phosphate. SPS enzymes are frequently encoded as a bi-domanial protein that also contains sucrose-6-phosphate phosphatase (SPP) activity that dephosphorylates this intermediate to sucrose. We and others have found that expression of sucrose permease (*cscB*), a symporter of sucrose and protons, will lead to the export of cytosolic sucrose to the medium (Ducat et al., 2012; Santos-Merino et al., 2023). In *S. elongatus* PCC 7942 strains with inducible copies of both *sps* and *cscB*, sucrose synthesis and export can be achieved in the absence of external osmotic pressure (Abramson et al., 2016, 2018; Lin et al., 2020; Dan et al., 2022). We therefore selected this strain to minimize the requirement for addition of salt to the growth medium.

**Figure 1:**
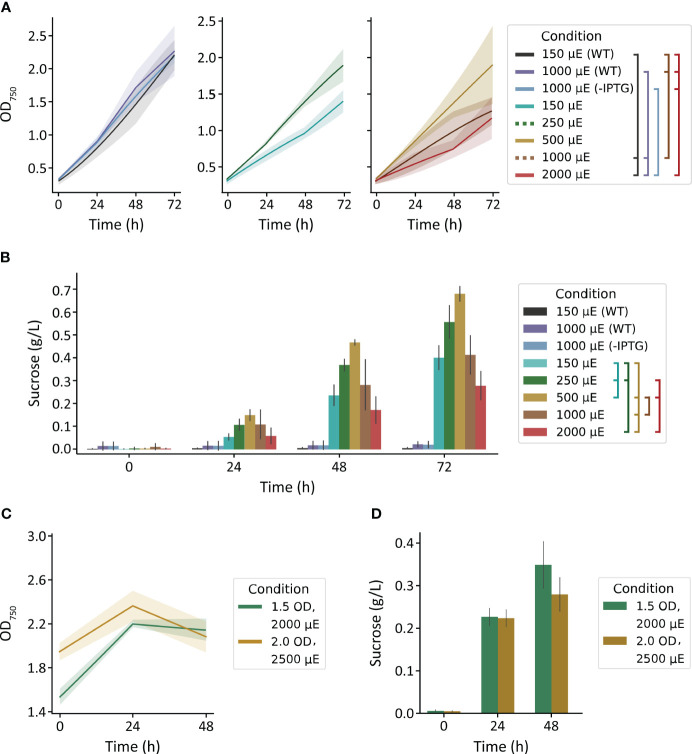
Growth and sucrose yields of WT and *cscB*/*sps*-expressing *S. elongatus* grown in the MC-1000 under various light intensities. **(A)** The OD_750_ of non-sucrose exporting (center and right) strains. Plots were split for visual clarity. **(B)** sucrose production of cultures started at low cell density (~0.3 OD_750_). Brackets in the legend denote significant differences (p < 0.05) at 72 h by one-way ANOVA in **(A)** all cultures and **(B)** induced cultures. The **(C)** OD_750_ and **(D)** sucrose production of cultures started at high cell densities (~1.5 and 2 OD_750_). Error bars denote standard deviation.

We first used the MC-1000 multi-cultivator to evaluate the impact of varied light intensity (150, 250, 500, 1000, 2000 µmol photons m^-2^ s^-1^) on growth and sucrose production of *S. elongatus* PCC 7942 *cscB*/*sps* strains and to broadly assess light tolerance. The growth of WT strains cultured with 150 and 1000 µmol photons m^-2^ s^-1^, and non-exporting *cscB*/*sps* with 1000 µmol photons m^-2^ s^-1^ grew at similar rates, reaching a final density of ~2.2 OD_750_ (**Figure 1A**, left). Sucrose-exporting strains, however, can partition a large fraction of their fixed carbon to secreted sucrose, leading to less cellular growth and biomass under certain conditions (Ducat et al., 2012)(*see also*, **Supplementary Figure S3**). The highest OD_750_ among sucrose exporters were from cultures grown with moderate irradiances of 250 and 500 µmol photons m^-2^ s^-1^ (**Figure 1A**, center and right), though differences were not statistically significant from other exporting conditions (p > 0.05). For sucrose-secreting lines in the MC-1000, there was a direct correlation between illumination and both cell growth and sucrose production up to 500 µmol photons m^-2^ s^-1^; light intensities beyond this threshold led to a decline in productivity (**Figures 1A, B**). Sucrose production over 72 hours peaked at 0.56 g L^-1^ for 250 µmol photons m^-2^ s^-1^ and 0.68 g L^-1^ for 500 µmol photons m^-2^ s^-1^ (p < 0.001 against all other light conditions) (**Figure 1B**). The relationship between irradiance and growth, however, is abolished when strains are not induced to export sucrose, and growth rates were relatively unaffected by illumination between 150 and 1000 µmol photons m^-2^ s^-1^ (**Figure 1A**, left). These data suggest that high light may indeed be harmful, and the increased photosynthetic capacity provided by a heterologous sink (*i.e.*, sucrose export) is not protective against light stress. However, it appears that *S. elongatus* PCC 7942 can remain resilient when not imposed with the additional burden of producing and secreting sucrose.

To evaluate if the sucrose productivity in these experiments was limited by a low-starting density, we increased the starting inoculation density. Since the effects of self-shading within higher density cultures lead to an effectively lower irradiation under the same outside illumination, we utilized brighter lights for our high-density starter cultures of 1.5 and 2.0 OD_750_, the upper range of OD_750_ the MC-1000 was able to support. To select the irradiances for these dense cultures, we revisited the most successful light intensity in our previous experiment. Cultures of 0.3 OD_750_ exposed to 500 µmol photons m^-2^ s^-1^ light receive ~300 µmol photons m^-2^ s^-1^ light when measured 1 cm from the surface (**Supplementary Figure S4**). Application of 2000 and 2500 µmol photons m^-2^ s^-1^ to cultures of 1.5 and 2.0 OD_750_, respectively, would achieve similar internal light intensities (**Supplementary Figure S4**).

We observed limited growth within the first 24 h of these cultures, but the density of the culture plateaued or declined in the following day to values statistically similar to starting turbidities (p > 0.05) (**Figure 1C**). Cultures grown with 2000 µmol photons m^-2^ s^-1^ produced 0.23 g L^-1^ sucrose within the first day, and had a final sucrose content of 0.35 g L^-1^ (p > 0.4); cultures grown with 2500 µmol photons m^-2^ s^-1^ produced 0.22 and 0.28 g L^-1^ sucrose at 24 and 48 h (p = 1.0), respectively (**Figure 1D**). Notably, the level of sucrose production from high density cultures was only marginally improved (~15%) at 48 h relative to cultures seeded at a lower density grown with 2000 µmol photons m^-2^ s^-1^ (p = 0.002) (**Figures 1B, D**).

### 3.2 Optimizing high cell density cultivation with increased irradiance and CO_2_

Curiously, both the cellular growth rate and sucrose productivity were relatively modest in the MC-1000 in comparison to values reported for similar strains by our group and others (Santos-Merino et al., 2023), suggesting that these cultures might be limited by another environmental variable (**Figures 1A, B**). Although 3% CO_2_ was bubbled into the cultivation tubes for these experiments, the method of delivery (sparging via a large-bore needle) may not be the most efficient compared to other methods for enriching inorganic carbon. Consistent with a possible inorganic carbon limitation, sucrose productivity was slightly, but significantly, enhanced in MC-1000 cultures supplemented with 10 mM HCO_3_^-^ (**Figure 1B**) relative to cultures without bicarbonate when grown with 500, 1000, and 2000 µmol photons m^-2^ s^-1^ (p = 0.038, 0.020, 0.028, respectively) (**Supplementary Figure S5**). However, we were unable to overcome any potential limitation of inorganic carbon in dense cultures through the supplementation of additional HCO_3_^-^ (**Supplementary Figure S6**).

**Figure 2:**
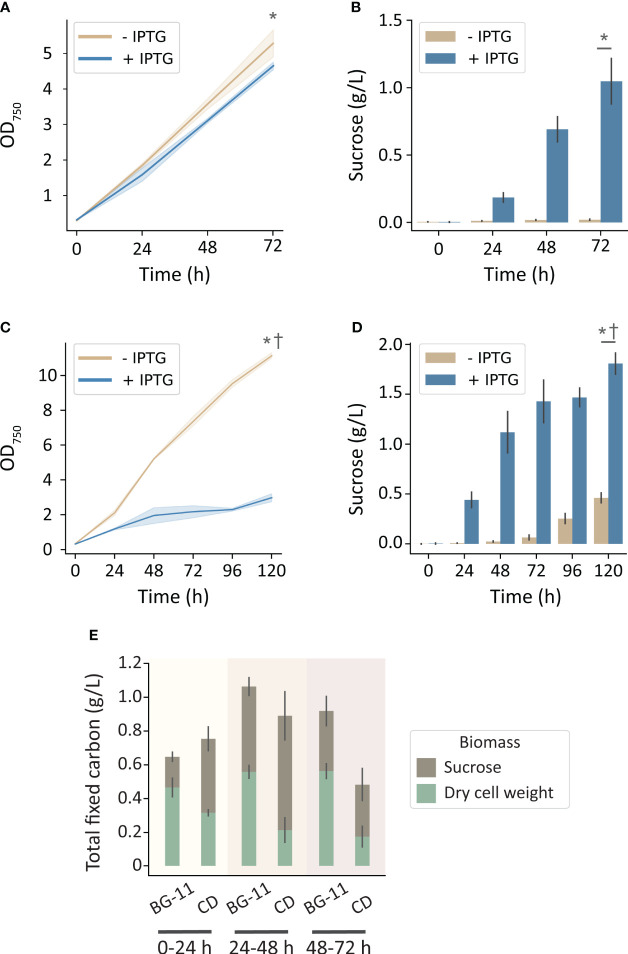
Growth and sucrose production of induced and uninduced *cscB*/*sps*-expressing *S. elongatus* grown in the CellDEG HDC cultivator system at moderate light intensity (~250 μmol photons m^-2^ s^-1^). The OD_750_ and sucrose production, respectively, of cultures grown in **(A, B)** BG-11 media, and **(C, D)** CD media. **(E)** Total fixed carbon for induced strains in BG-11 and CD media as shown by sucrose and dry cell weight accumulated per day. Error bars denote standard deviation. Asterisks denote significant differences between induction conditions at the indicated timepoint by independent samples t-test (p < 0.005). Daggers denote significant differences in induced cultures between **(A, B)** and **(B, C)** conditions by independent samples t-test (p < 0.001).

To more directly evaluate the hypothesis that carbon limitation was limiting for growth and sucrose productivity in the MC-1000, we utilized the High-Density Cultivation (HDC) system (CellDEG GmbH, Germany). These cultivation vessels are designed to deliver a saturating level of inorganic carbon through a permeable membrane with a large surface area (Lippi et al., 2018). Using an identical growth medium (BG-11), a significant enhancement of cell growth and sucrose production was observed in the CellDeg HDC system relative to the MC-1000 with comparable illumination (250 μmol photons m^-2^ s^-1^). Strains that were not induced to export sucrose grew to 5.3 OD_750_ over 72 h while producing a marginal amount of sucrose (0.02 g L^-1^). Strains induced to secrete sucrose grew to 4.6 OD_750_ and produced 1.0 g L^-1^ sucrose (**Figures 2A, B**). Curiously, under these conditions of high inorganic carbon availability, we observed little, but significant, difference in growth rate in the sucrose-exporting strains despite a significant rate of carbon export in the form of sucrose (**Figures 2A, B, E**).

Since nutrients in BG-11 medium can become limiting at higher density, we next evaluated an enriched medium (CD) designed to support high-density cultures, such as those achievable in the HDC system (Dienst et al., 2020). When cultures were grown in CD media, non-exporting strains reached 11.1 OD_750_ over the course of 5 days (**Figure 2C**), while also secreting a disproportionally high level of sucrose for an uninduced culture (**Figure 2D**). Strains induced to export sucrose were significantly stunted in growth after 120 h (3 OD_750_; **Figure 2C**) and secreted sucrose at a higher rate.

### 3.3 Increasing light and CO_2_ availability to enhance sucrose synthesis

**Figure 3:**
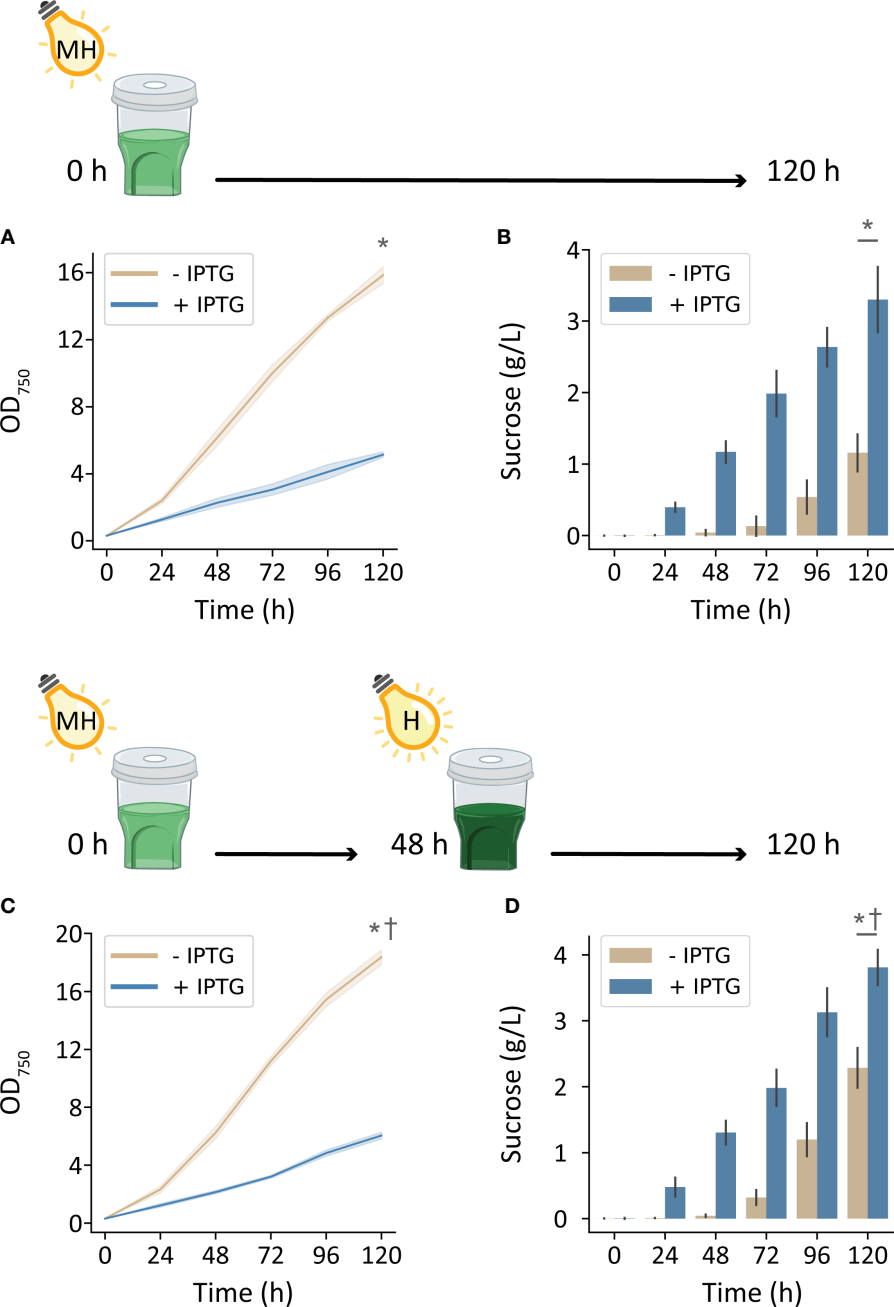
Growth and sucrose production of induced and uninduced *cscB*/*sps*-expressing *S. elongatus* grown in the HDC system with moderately high and high-light intensity (MH and H, respectively) and CD media. The OD_750_ and supernatant sucrose concentration of cultures grown with **(A, B)** 500 μmol photons m^-2^ s^-1^ for 0-120 h, and **(C, D)** 500 μmol photons m^-2^ s^-1^ for 0-48 h and 1000 μmol photons m^-2^ s^-1^ for 48-120 h. Error bars denote standard deviation. Asterisks denote significant differences between induction conditions at the indicated timepoint by independent samples t-test (p < 0.001). Daggers denote significant differences in induced cultures between **(A, B)** and **(C, D)** conditions by independent samples t-test (p < 0.005).

Environmental conditions enriched with nutrients and inorganic carbon supported substantially higher cyanobacterial production with increased irradiation. Cultivating *cscB*/*sps* strains with high CO_2_ (HDC) and with 500 μmol photons m^-2^ s^-1^, non-exporting and sucrose-exporting strains reached OD_750_ of 15.8 and 5.2, respectively (**Figure 3A**). Furthermore, strains induced to secrete sucrose accumulated up to 3.3 g L^-1^ over the course of 5 days, while uninduced cultures accumulated 1.15 g L^-1^ (**Figure 3B**). Further enhancements in bioproduction could be achieved by further increasing light intensity to 1000 μmol photons m^-2^ s^-1^ at 48 h when cultures were more turbid: sucrose-secreting strains reached an OD_750_ of 6.0 and sucrose yields of 3.8 g L^-1^ (**Figures 3C, D**). Uninduced strains under these growth conditions achieved 18.4 OD_750_ and secreted a surprisingly high amount of sucrose, reaching 2.3 g/L sucrose at the end of 120 h.

## 4 Discussion

Considerable variation in the productivity of cyanobacteria engineered to secrete sucrose has been reported in the literature (a summarized table can be viewed in Santos-Merino et al., 2023);, and this variability has significant implications for the viability of biotechnological applications. Obviously, light is a crucial component of photosynthetically driven cultivation, yet it is also well-established that over-irradiation of cyanobacteria can lead to cell damage and/or death if surplus light energy is not safely dissipated (Montgomery, 2014). *S. elongatus* PCC 7942 has been often described in the literature as a strain that prefers a relatively low-light, and many groups routinely cultivate this strain ≤100 µmol photons m^-2^ s^-1^. Our results show that light and CO_2_ availability strongly impact the bioproductivity of sucrose-secreting cyanobacterial strains, independent of the genetic background. Under conditions of high nutrient and CO_2_ availability, cell biomass and sucrose secretion rates are positively correlated with irradiation, up to 1000 μmol photons m^-2^ s^-1^ (**Figures 2**, **3**): a light intensity regarded as photodamaging for *S. elongatus* PCC 7942 in some literature. The light tolerance we observe is consistent with recent publications reporting that the maximum growth rate of *S. elongatus* PCC 7942 is achieved with 400 µmol photons m^-2^ s^-1^ (Takatani et al., 2015; Ungerer et al., 2018a). Further complication of *S. elongatus* PCC 7942’s light tolerance is that self-shading in dense cultures or chambers with long optical cross-sections can rapidly attenuate effective light availability (Andersson et al., 2019). Our results suggest that differences in cyanobacterial cultivation conditions likely can account for a significant portion of variability (up to 3- to 10-fold) in reported sucrose production values, even when solely accounting for strains grown in common, commercially available photobioreactors.

We observe that the mechanism of CO_2_ delivery strongly impacts the bioproduction potential for sucrose-secreting cyanobacteria at higher light intensities. In the MC-1000 cultivator, CO_2_ is delivered via a bubble column-style sparging system, and we observed indications of carbon limitation despite continual sparging with 3% CO_2_ gas. In the MC-1000, a positive correlation for production was observed with increasing illumination up to ≤ 500 μmol photons m^-2^ s^-1^ (**Figures 1A, B**). Interestingly, even the highest light conditions were not lethal (**Figure 1A**; 2000 µmol photos m^-2^ s^-1^ is roughly equivalent to full, midday sunlight), although *S. elongatus* PCC 7942 is frequently described in the literature as a low-light tolerant strain (*e.g*., Ungerer et al., 2018b; Takatani et al., 2022; Walker and Pakrasi, 2022). Furthermore, the final OD_750_ and sucrose productivities of strains cultivated in the MC-1000 system were substantially lower than values reported by our group and others with the same growth medium in other bioreactor designs (Abramson et al., 2016, 2018; Qiao et al., 2018). One possible explanation may be that inorganic carbon availability could be limiting, despite the continual sparging of 3% CO_2_ delivered via a stream of CO_2_ bubbles through a submerged needle design (Markou et al., 2014).

When cultures were grown under conditions with greater inorganic carbon and/or nutrient availability, increasing illumination resulted in an even greater boost in productivity. The HDC system uses cultivators with a gas-permeable bottom inserted into a carrier plate that exposes it to the CO_2_ headspace created by a HCO_3_^-^ buffer reservoir; the device can generate up to 8% CO_2_ under the conditions outlined in this work (CellDEG GmbH, 2020). Using the HDC system, uninduced *cscB*/*sps* strains achieved an OD_750_ of 5.3, while induced strains reached an OD_750_ of 4.6 and sucrose yield of 1.0 g L^-1^ when grown in BG-11 media (**Figures 2A, B**). For sucrose-exporting strains, this amounts to 245% higher OD_750_ and 188% more sucrose than cultures grown in the MC-1000 with the same media and irradiance (250 µmol photons m^-2^ s^-1^). Importantly, under conditions of saturating inorganic carbon, we do not observe a “cost” for sucrose secretion, as indicated by the comparable levels of cellular biomass even when substantial sucrose is secreted (**Figure 2E**). This suggests that growth does not have to be sacrificed when inorganic carbon is not a limiting factor, assuming that the fraction of fixed carbon diverted to sucrose production does not exceed a threshold value. By comparison, when grown in CD media, the OD_750_ of non-exporting strains rose to an average of 11.1, but the OD_750_ of sucrose-secreting strains dropped to ~3.0 at the end of the 120 h experimental period (**Figure 2C**). We attribute this loss of cell biomass in cultures induced to secrete sucrose to the higher osmotic pressure of CD medium. At ~140 mOsm, CD medium has an osmolarity that is approximately 3-fold higher than that of BG-11 alone and can provide osmotic pressure equivalent to BG-11 supplemented with ~50 mM NaCl. The strain in this study produces sucrose utilizing a heterologous salt-independent SPS; however, increased salt or osmotic stress engages the endogenous salt-dependent sucrose production pathways and has been shown to lead to a higher diversion of fixed carbon toward sucrose biosynthesis (Ducat et al., 2012; Kirsch et al., 2019; Liang et al., 2020). Sucrose exporters grown in CD media produced 139% more sucrose than their BG-11 counterparts by 72 hours, and a total of 1.8 g L^-1^ sucrose by 120 h (**Figure 2D**). Non-exporting strains in CD media produce more sucrose than in BG-11 as well; this can also be explained by the medium exerting osmotic stress, and thus activating the native SPS.

To potentially combine the productivity gains of cultivating with high light with those of high CO_2_ availability, we required a separate light source capable of reaching intensities beyond those of most commercial photobioreactors. For this purpose, we created a custom LED light source in-house consisting of four COB LEDs used for growth of photosynthetic organisms; when placed above the HDC and shaker, there is generally an equitable distribution of light between the six cultivators. By cultivating strains in the HDC system with CD media and 250 µmol photons m^-2^ s^-1^, growth and sucrose yield increased in both induced and uninduced *cscB*/*sps* strains. By doubling irradiance to 500 µmol photons m^-2^ s^-1^, OD_750_ and sucrose yield increased by 173% and 182%, respectively, in *cscB*/*sps* expressing strains (**Figures 3A, B**). Non-sucrose exporting strains underwent a 142% and 251% increase in growth and sucrose, respectively. Finally, a further increase in culture productivity could be achieved by both increasing light intensity to 1000 µmol photons m^-2^ s^-1^ while taking advantage of the self-shading of higher density cultures. By doubling the light intensity of cultures that were grown with 500 µmol photons m^-2^ s^-1^ at 48 h (average of 2.3 OD_750_), cell growth increased by 17% (p < 0.001) and sucrose productivity increased by 15% (p < 0.005) (**Figures 3C, D**). The highest sucrose productivity we report was reached under these conditions, at 47.8 mg L^-1^ h^-1^. Surprisingly, sucrose bioproduction was nearly as strong in strains not induced to express *cscB*/*sps* (highest productivity observed at 45.3 mg L^-1^ h^-1^), likely due to a combination of very high cell density (OD_750_ up to 18.3) and induction of endogenous sucrose production via native osmotic-responsive pathways (**Figures 3A, B**). This latter observation may suggest an alternative route for high sucrose production from late-phase, dense cultures with only basal levels of activation of the sucrose export pathway.

## 5 Conclusion

In this study we assessed how irradiance, inorganic carbon availability, and cultivation method affect the growth and sucrose production of *S. elongatus* PCC 7942. We found that increasing light intensity up to 1000 µmol photons m^-2^ s^-1^ has a positive effect on sucrose production, and higher irradiances can be tolerated if cultures grow into sufficient turbidity. By adjusting light regimes and increasing CO_2_ availability, *S. elongatus* PCC 7942 *cscB*/*sps* reached a productivity of 47.8 mg sucrose L^-1^ h^-1^, which is comparable or exceeds other reported strains, including some metabolic engineering interventions that can negatively affect cell health. Our survey underscores the importance of cultivation conditions on sucrose productivity and may partially explain the variability observed across different institutions using similar strains. These results may contribute insights into future optimization of cyanobacterial bioproduction efforts.

## Data availability statement

The original contributions presented in the study are included in the article/**Supplementary Material**. Further inquiries can be directed to the corresponding author.

## Author contributions

LY: Data curation, Formal analysis, Investigation, Methodology, Project administration, Resources, Validation, Visualization, Writing – original draft, Writing – review & editing. RZ: Resources, Writing – review & editing. DCD: Conceptualization, Funding acquisition, Methodology, Project administration, Supervision, Writing – original draft, Writing – review & editing.
