# Supplementary material for: Impact of irradiance and inorganic carbon availability on heterologous sucrose production in Synechococcus elongatus PCC 7942
Source: Front Plant Sci. 2024 Apr 8;15:1378573. doi: 10.3389/fpls.2024.1378573 (PMC11033428; doi:10.3389/fpls.2024.1378573)
Supplement: Supplementary file 1 [file Image_1.pdf]

frontiers

Supplementary Material

Supplementary Figures

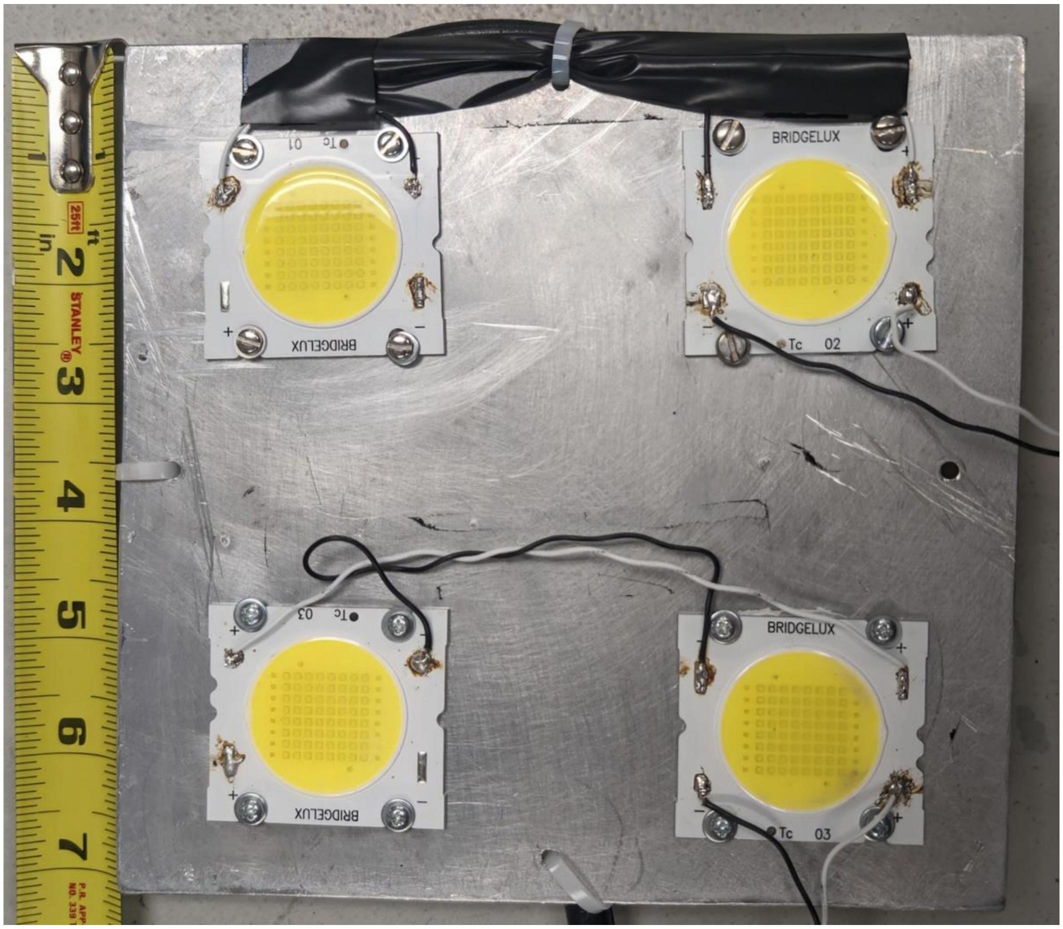

Supplementary Figure 1. Custom lighting array consisting of four COB LED lights (BXRA-56C5300-H, Bridgelux, USA) mounted on an aluminum panel two inches apart and connected to a power supply (LS35-24, TDK-Lambda, Japan). The panel was attached to a ring clamp, and the clamp fastened to a ring stand. Light intensity can be set by adjusting the distance of the panel to the HDC, or by modulating the voltage of the power supply.

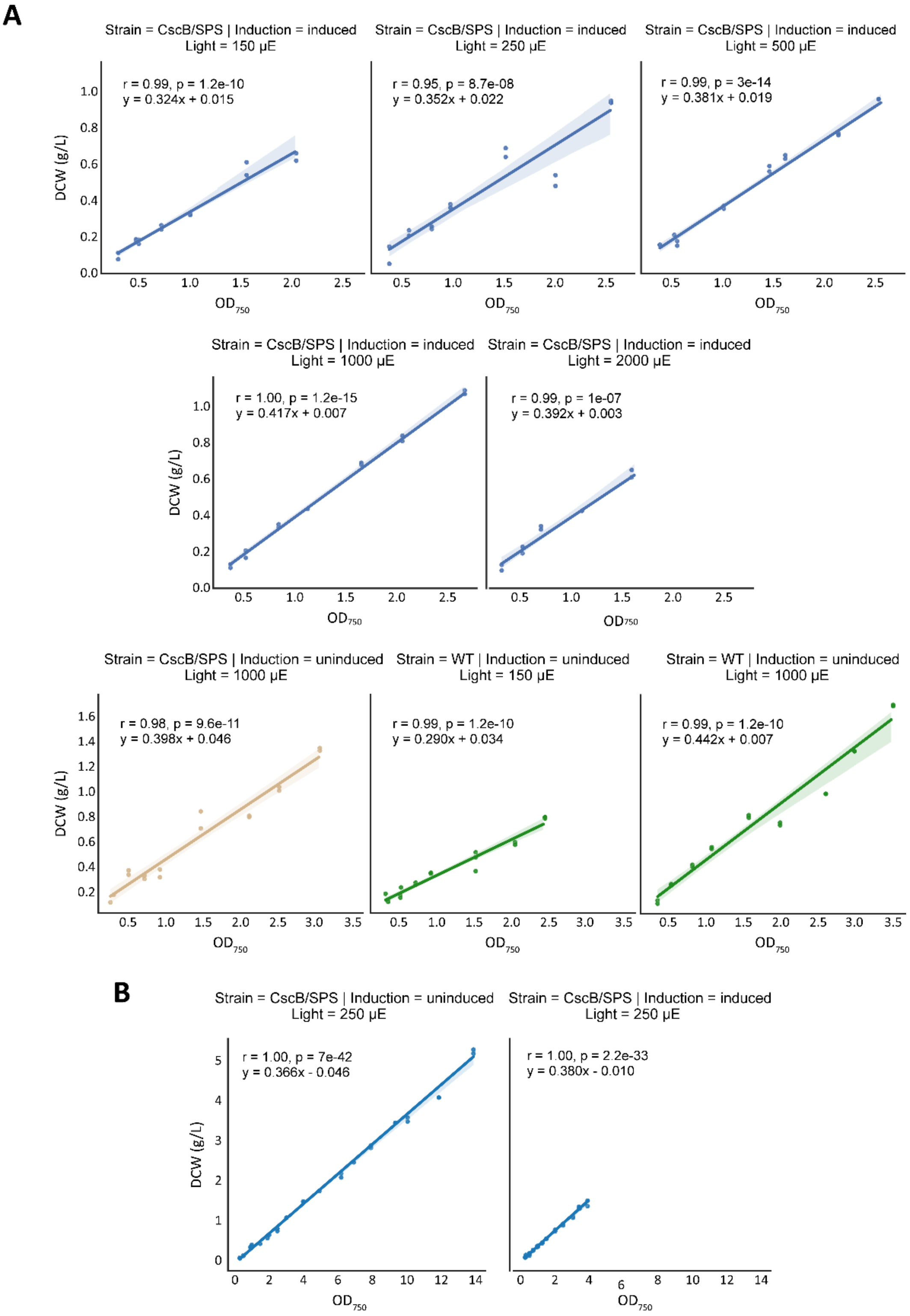

Supplementary Figure 2. Relationship between dry cell weight (DCW, g/L) and OD_{750} in different growth conditions and cultivators. **(A)** WT, and uninduced and induced *cscB*/*sps* cultured in the MC-1000 under various light intensities. **(B)** Uninduced and induced *cscB*/*sps* grown in the CellDEG HDC system with 250 µmol photons m^{-2} s^{-1} and 2% CO_2.

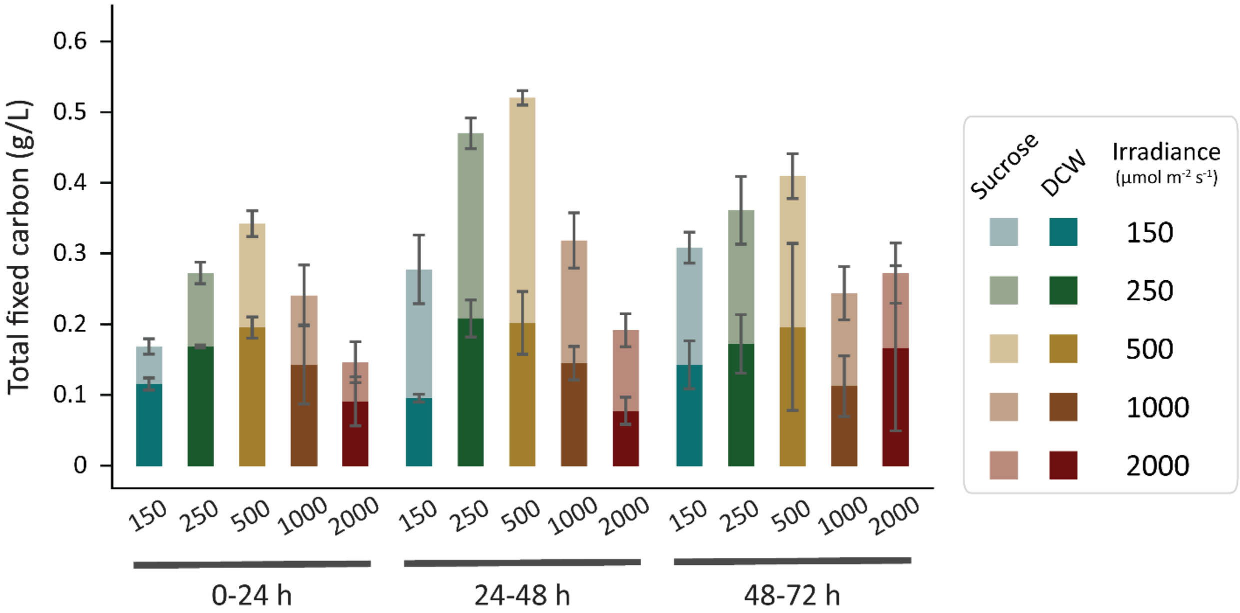

Supplementary Figure 3. Total fixed carbon as shown by sucrose and dry cell weight accumulated per day in cultures grown in the MC-1000 under varying irradiances. Error bars denote standard deviation.

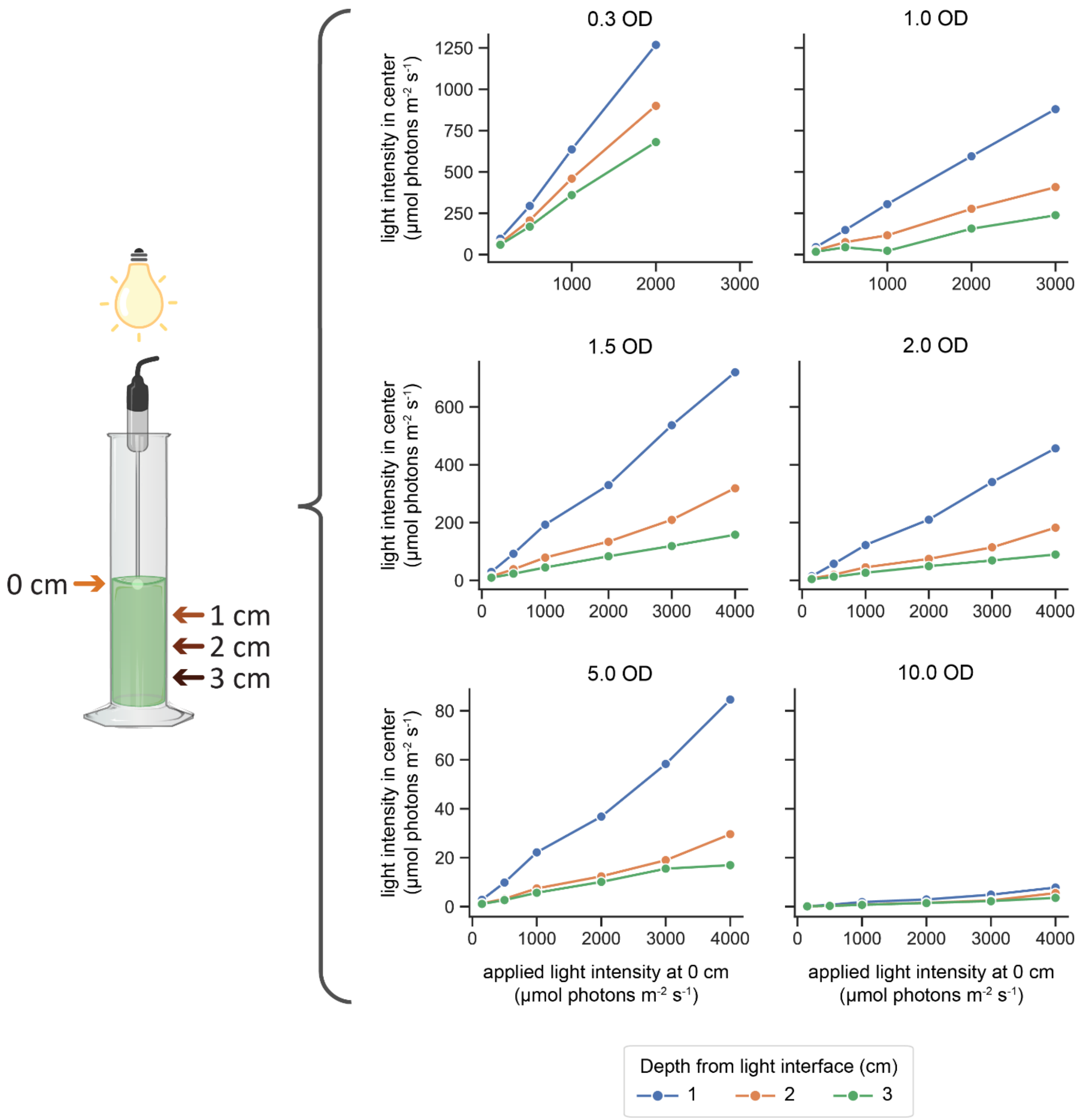

Supplementary Figure 4. Light perceived in the center of a culture at varying OD_{750}, distance from the light interface, and irradiance. Measurements are averaged values from a 15-second interval.

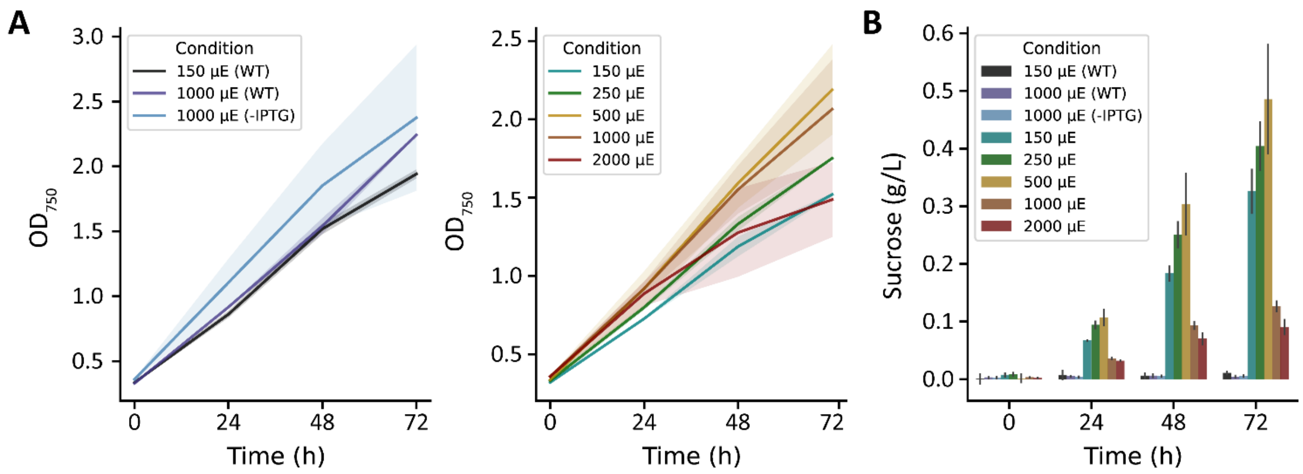

Supplementary Figure 5. Growth and sucrose production of WT and c*scB/sps*-expressing *S. elongatus* grown in the MC-1000 under various light intensities and 0 mM HCO_3^-. **(A)** OD_{750} and **(B)** sucrose production of cultures. Error bars denote standard deviation.

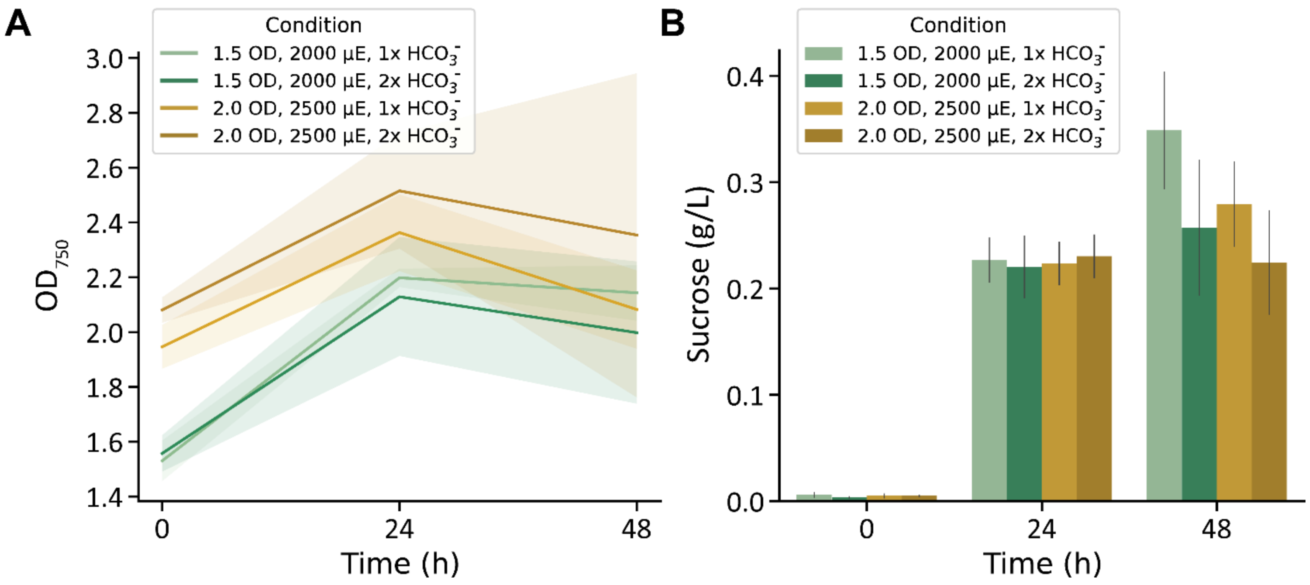

Supplementary Figure 6. (A) Growth as OD_{750} and **(B)** sucrose production of *cscB*/*sps*-expressing *S. elongatus* of cultures started at high cell densities (1.5 and 2 OD_{750}). Error bars denote standard deviation.
